# Genetic Characteristics of Three Single-Farm-Isolated Porcine Reproductive and Respiratory Syndrome Viruses with Novel Recombination among NADC30-Like, JXA1-Like, and QYYZ-Like Strains

**DOI:** 10.1155/2023/8871321

**Published:** 2023-07-22

**Authors:** Xiaoyu Cao, Xinna Ge, Yongning Zhang, Xin Guo, Jun Han, Lei Zhou, Hanchun Yang

**Affiliations:** ^1^National Key Laboratory of Veterinary Public Health and Safety, College of Veterinary Medicine, China Agricultural University, Beijing, China; ^2^Key Laboratory of Animal Epidemiology of Ministry of Agriculture and Rural Affairs, College of Veterinary Medicine, China Agricultural University, Beijing, China

## Abstract

Porcine reproductive and respiratory syndrome virus (PRRSV) is an economically devastating pathogenic microorganism that greatly affects the pork industry in the world. The genetic variation and frequent emergence of novel strains greatly hinder the control efforts of PRRSV. Therefore, monitoring the evolutionary dynamics is long and rewarding work for PRRSV researchers and practitioners to make the control strategy. Here, three novel PRRSV strains named CHbj2101, CHbj2102, and CHbj2103 were isolated from different nursery barns with various mortality rates from 6% to 17%, belonging to the same farm, but at different periods of the outbreak. The genomic sequencing and phylogenetic analyses indicated that these three isolates were all clustered with NADC30-like virus CHsx1401, sharing higher genomic similarity from 87.3% to 89.8%, and having the same molecular marker of 131 amino acid residues deletion at their nsp2 coding region, but varied mutagenesis among the antigenic sites in the region of GP2 to GP5. Among the available PRRSV sequences in the GenBank, the isolates CHbj2101 and CHbj2102 display the highest genomic identity (90.1% and 89.8%) with NADC30-like recombinant strain 15LN3, and the rest CHbj2103 shows the highest genomic identity (90.8%) with NADC30-like virus strain 15SC3. The recombination analysis indicated that all three isolates are generated by multiple recombination events among the NADC30-like virus (major parent, Lineage 1), HP-PRRSV vaccine-like virus (minor parent, Lineage 8), and QYYZ-like virus (minor parent, Lineage 3). The isolates CHbj2101 and CHbj2102 shared a similar recombination pattern, but CHbj2103 has a different pattern in nonstructural protein coding regions. To further investigate the recombination characteristics of QYYZ-like strains, we analyzed all available whole genomic sequences of QYYZ-like PRRSV, submitted during the year 1991 and 2021 (*n* = 83) in China. The result shows that almost all QYYZ-like strains were products of recombination and their immunogenicity or protective protein fragments (nsp2–nsp7 and GP2–GP4) were mainly from QYYZ. These results provide us with some better insight into the evolution process of PRRSV strains in the field and warn us to pay more attention to monitoring and reducing the PRRSV variant on farms to reduce the risk of novel emergence and outbreak.

## 1. Introduction

Porcine reproductive and respiratory syndrome (PRRS) is a viral disease characterized by reproductive failure in breeding animals and respiratory disorder affecting pigs of all ages [1]. PRRS initially emerged in both North America and Europe in the late 1980s, referred to “mystery” disease as it was initially confused with several other diseases, and it rapidly spread to Asia during the subsequent decade [2–4]. More than 30 years later, PRRS is still one of the biggest challenges for the global swine industry, causing serious economically damaging [5–7]. The causative pathogen PRRS virus (PRRSV), belongs to the genus Porartevirus of the family Arteriviridae in the order Nidovirales [8, 9]. Recently, the two species PRRSV-1 (type 1) and PRRSV-2 (type 2) have been classified as Betaarterivirus suid 1 and Betaarterivirus suid 2, respectively, in the genus Betaarterivirus [10]. There was about only 55%–70% nucleotide identity between these two species. According to the phylogenic analysis of ORF5, PRRSV can be divided into nine distinct genetic lineages within PRRSV-2, and three subtypes within the PRRSV-1, at least [11].

PRRSV is an enveloped virus, with a single-stranded and positive-sense RNA genome, which is approximately 15 kb in length, consisting of a 5′-untranslated region (UTR) and a 3′UTR with a poly (A) tail at both terminals, as well as 10 overlapping open reading frames (ORFs) [12]. ORF1a and ORF1b encode two polymerase proteins, which can be cleaved into at least 14 nonstructural proteins (nsps) involved in PRRSV genome replication and RNA transcription. The rest ORFs encode eight structural proteins, including glycoproteins GP2, GP3, GP4, GP5, GP5a, and nonglycoproteins E, M, and N [13]. The GP2–GP5, as well as M and E, are the main viral envelope proteins related to cell adsorption, entry, and neutralization [14].

The rapid mutability and high-evolution rate drive PRRSV to be a “sneaky” virus with extensive genetic and antigenic variation, which is regarded as a strategy for escaping from host immune response. This diversity was initially attributed to the introduction of new strains into farms, pig movement, and diversification by site mutations and virus recombination. Recombination between two different strains has been demonstrated in early studies through both cell and pig coinfection experiments [15, 16]. Increasingly, clinical evidence has suggested that recombination contributes more genetic diversity and quick evolution to the PRRSV genome [17, 18]. Noticeably, the usage of the modified live virus (MLV) vaccine might also enhance strain diversity. Since the first commercial MLV was available on the market, it has been widely used for more than 20 years and has a remarkable contribution to increasing the production performance of the pig. However, it also raises some concerns, as MLV can still cause infection in the pig, leading to replication, and viral shedding, meanwhile, it fails to achieve sterilizing immunity and clean the virus immediately, which increases the risk of virulence reversion and recombination with coinfected other strains. Meanwhile, the immune selection pressure from vaccination might also accelerate viral mutation and recombination [19]. These variations are leading to a more complex PRRSV epidemic situation in the field.

China is the leading pork producer worldwide, raising more than 50% of the global pig population. Various PRRSV strains have caused outbreaks and epidemics in China. In 1995, the outbreak of PRRS was first reported on an intensive pig farm in northern China. In the following years, PRRSV quickly spreads from the north to the most pig-raising area of this country and became highly prevalent in China [20]. In 2006, a novel highly pathogenic PRRSV (HP-PRRSV) strain began to emerge in China, which brought an unparalleled threat to commercial pork production in the subsequent years [21, 22]. Then, the HP-PRRSV-derived commercial MLVs, including JXA1-R (JXA1-P80), HUN4-F112, and TJM have been broadly used for PRRS prevention. Since 2011, more and more PRRSV isolates have been identified to genetically close to the United States Lineage 1 strain NADC30, then this kind of strain, named NADC30-like virus, gradually become the predominant PRRSV strain in China [23]. Another kind of Lineage 1 strain, the NADC34-like virus, which was first isolated in China in 2017, has been often isolated from the field since 2020 [24, 25]. Finally, the QYYZ-like virus from Lineage 3 was initially reported in mainland China in 2005, and then they were sporadically found on farms in central and southern China [26]. Considering the limited heterologous protection among different kinds of strains, this variety seriously increased the difficulty of PRRSV control in China.

In this study, three PRRSV isolates were obtained from the same pig farm in China, then the phylogenetic and recombination analyses were performed. The results indicated that the three PRRSV isolates were generated from multiple recombination events among Lineage 1 NADC30-like virus as the major parent, both Lineage 8 HP-PRRSV vaccine-like virus and Lineage 3 QYYZ-like virus as the minor parent. To further investigate the recombination characteristics of the QYYZ-like virus, all available full-genome sequences of QYYZ-like PRRSV (isolated between 1991 and 2021) in GenBank, were also analyzed to exlore a panoramic view of their recombination. The work will provide some new insights into the PPRSV evolutionary dynamics, especially for the recombination preference of QYYZ-like viruses.

## 2. Materials and Methods

### 2.1. Samples and Viruses

Lung and inguinal lymph node samples from five weaning pigs were collected on an intensive breading pig farm (with 1,000 head sows) in Beijing, where serious respiratory disorder was observed in the herd. The collected lymph nodes and lung samples were mixed with a 1 mL Roswell Park Memorial Institute (RPMI) 1640 Medium (Thermo Fisher Scientific, Waltham, MA, USA), and ground in the TissueLyser (TissueLyser2, Qiagen, Germany). After centrifugation, the supernatant was passed through a filter with pores of 0.22 *μ*m to remove the bacteria. The PRRSV target cell, porcine alveolar macrophages (PAM) were obtained from 30-day-old specific-pathogen-free (SPF) pigs for isolating PRRSV from the collected samples. When PRRSV-specific cytopathic effect (CPE) can be observed from approximately 80% of virus-inoculated cells, the virus was harvested after freeze-thawing once and centrifuging for 15 min at 4°C, with the speed of 8,000 rpm, then the supernatant was collected for further passages or stored at −80°C [27]. The isolated virus was passaged in PAM cells three times. After that, the virus was identified by using indirect immunofluorescence assay (IFA) with monoclonal antibody (mAb) SDOW-17 (Rural Technologies, Inc., Brookings, SD, USA) against the PRRSV N protein [28].

### 2.2. RT-PCR and Sequencing

The viral RNAs, extracted from tissue samples or harvested supernatants of virus-inoculated cells, by using TRIzol™ Reagent (Thermo Fisher Scientific, Waltham, MA, USA), were used for reverse transcription polymerase chain reaction (RT-PCR) with FastKing Reverse Transcription Kit (with gDNase) (TIANGEN, Beijing, China), KOD DNA polymerase (TOYOBO, Shanghai, China) and 14 pairs of PRRSV-specific primers designed in the previous study [29]. UTRs were amplified by using 5′ and 3′ Full RACE kits (TaKaRa, Dalian, China), as the instructions provided by the manufacturer. All RT-PCR products were purified by the EasyPure® Quick Gel Extraction Kit (Transgen, Beijing, China), and submitted to Tsingke Biotech Co., Ltd. (Beijing, China) for Sanger sequencing. Finally, the sequences of all fragments were assembled by the program of Seqman from DNASTAR 7.1 software (DNASTAR Inc., Madison, USA).

### 2.3. Phylogenetic Analysis and nsp2 Deletion Patterns

To study the evolution process of the isolated PRRSV strains, 57 representative PRRSV genomic sequences from various lineages were acquired from the GenBank database as reference sequences (Table S1). Based on the sequences of the nsp2, ORF5, ORF7, and whole genomes, phylogenetic trees were drawn by utilizing the neighbor-joining method in MEGA 7.0 with a bootstrap value of 1,000 replicates. And the PRRSV strains were classified into different lineages based on the ORF5 sequences [30].

To identify the patterns of nsp2 deletion in these isolates, amino acid sequences of nsp2 were aligned among the isolates and the representative strains (VR2332, JXA1, JXA1 P80, NADC30, CHsx1401, NADC34, and LNWK130) by using ClustalW in the MegAlign.

### 2.4. N-glycosylation Analysis

The GP5 amino acid sequences of these three isolates were further aligned with other reference strains. On the online NetNGlyc 1.0 server, the putative N-glycosylation profiles of GP5 were predicted and presented (http://www.cbs.dtu.dk/services/NetNGlyc/).

### 2.5. Recombination Analysis of the Newly Isolated Strains

SimPlot software v.3.5.1 was applied to analyze the recombination events of these three isolates, in which the boot scanning process was executed with the step size and window size as 20 and 200 bp, respectively. To further identify the recombination events and estimate the positions of breakpoints, seven different algorithms from RDP 4.0 software, including 3Seq, RDP, MaxChi, Chimera, GENECONV, SiScan, and Bootscan were selected to identify the potential recombination signals and hot breakpoint locations in the genome of these three isolates. To ensure the recombination occurred in pigs, but not during cell culture, the flanking sequences of recombination breakpoints were amplified and submitted for resequence. The additional phylogenetic analysis based on the recombination regions was also carried out as previously described methods.

### 2.6. Interlineage Recombination Analysis of QYYZ-Like Strains

To further investigate the recombination pattern of the QYYZ-like PRRSV, all available QYYZ-like strains isolated from the year 1991 to 2021 in China were downloaded for recombination analysis. The strains were screened out as QYYZ-like virus, according to the following criteria, which was modified from a previous report [31]: (a) same as QYYZ, classified into the Lineage 3 in the ORF5-based phylogenetic tree; (b) sharing the highest ORF5 sequence identity with QYYZ (>85.8%); and (c) presenting the same amino acids (aa) “marker” unique to QYYZ derivatives. Nine representative parental strains from different lineages of PRRSV-2 were selected as references.

## 3. Results

### 3.1. PRRSV Isolation and Identification

**Figure 1:**
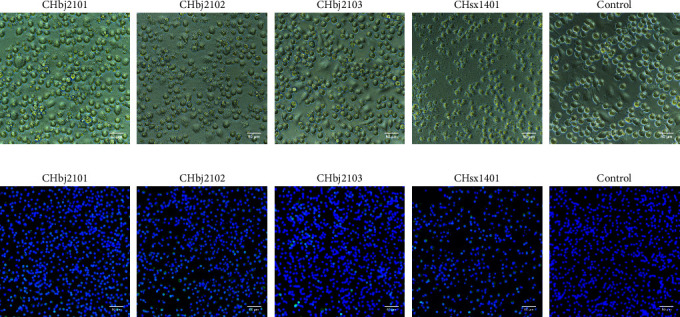
Identification of the three isolates in PAMs. (a) Cytopathic effect (CPE) induced by CHbj2101, CHbj2102, and CHbj2103 in PAMs were observed at 48 hpi, respectively. (b) The presence of isolates was identified by immunofluorescence assay (IFA). Cells were fixed and labeled by using an anti-N protein monoclonal antibody (green). NADC30-like CHsx1401 was treated as a positive control. Magnification, 200×.

The weaning pigs showed typical clinical signs of PRRS, including coughing, fever (up to 41°C), lethargy, and depression. The lung tissues and lymph nodes collected from five dead pigs were further confirmed to be PRRSV positive by RT-quantitative polymerase chain reaction (qPCR) carried out in the farm's diagnosis lab. In order to isolate the virus, the supernatant of ground tissue samples tested as PRRSV positive was used to inoculate PAMs. Meanwhile, the NADC30-like strain CHsx1401 was set as the positive control. The PRRSV-specific CPE can be observed on PAMs, which were inoculated with the CHsx1401 virus or the other three PRRSV-positive samples at the third passage (Figure 1(a)). The IFA results further confirm that PRRSV was successfully isolated from these three samples (Figure 1(b)) and designated as CHbj2101, CHbj2102, and CHbj2103, respectively.

### 3.2. Complete Genomic Sequencing and Analysis

Fourteen pairs of PCR primers were designed to apply the overlap fragments that covered the whole genomes of these three PRRSV isolates, and the 5′RACE and 3′RACE were also applied by using commercial kits. The applied fragments were submitted for sequencing via the Sanger method. The assembled whole-genomic sequences of these three isolates were uploaded to the GenBank database with the accession number CHbj2101-OP734316, CHbj2102-OP734317, and CHbj2103-OP734318, respectively. Excluding the poly (A) tail, the genomes of these three isolates were 15,016 nt in length, with a 187 nt-5′UTR and a 151nt-3′UTR. As shown in Table 1, these three PRRSV isolates share 90.9%–98.8% nucleotide identity among their genomes. The genomic sequences of CHbj2101 and CHbj2102 are much closer with a nucleotide identity of 98.8%. When compared with the representative reference PRRSV trains, these three isolates showed 85.1%–85.2% nucleotide identity with the PRRSV-2 prototype strain VR2332 (Lineage 5), 85.5%–86.3% with the HP-PRRSV strains (Lineage 8), and 83.8%–84.1% with QYYZ (Lineage 3). These three isolates show higher similarity to the NADC30-like virus CHsx1401 (87.3%–89.8%) than that to NADC34-like LNWK130 (83.6%–83.9%). Among the available PRRSV sequences in the GenBank, the isolates CHbj2101 and CHbj2102 show the highest genomic similarity (90.1% and 89.8%) with strain 15LN3, and the isolate CHbj2103 displays the highest genomic similarity (90.8%) with 15SC3. Both 15LN3 and 15SC3 have been reported to be clustered into Lineage 1, which contains NADC30-like virus and their recombinants [32].

### 3.3. Sequence Analysis of the nsp2 Coding Region

**Figure 2:**
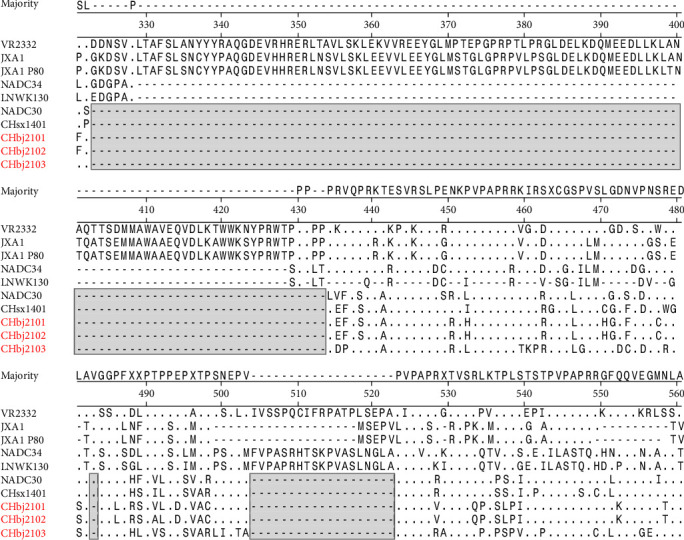
Alignment of the nsp2 amino acid sequence of the three PRRSV isolates with representative PRRSV strains. Deletions in the three isolates and related virus strains were shaded in gray.

PRRSV nsp2 exhibits significant variability. The specific insertion or deletion pattern in its hypervariable region is often regarded as a molecular marker for specific kinds of strains. To explore the sequence characteristics of the nsp2 coding region in these three isolates, their nsp2 sequences were aligned with representative strains from Lineage 5 (VR2332), Lineage 8 (JXA1, JXA1 P80), and Lineage 1 (NADC30, CHsx1401, NADC34, and LNWK130). Compared with the representative reference strains, the nucleotide and amino acid identities are 76.0%–88.7% and 71.7%–85.1%, respectively. All three isolates share the highest nsp2 similarity with NADC30 (82.4%–88.7% nucleotide identity and 85.1%–87.3% amino acid identity). Meanwhile, the nsp2 coding region of all three isolates have the 131-aa (111 + 1 + 19 aa) discontinuous deletions compared with VR2332, which was the “molecular marker” of the NADC30-like virus (Figure 2).

### 3.4. Sequence Analysis of the Major Glycoprotein GP5

The gene ORF5 encoding the major glycoprotein GP5 is another highly variated region of the genome. It has been widely used for the classification of lineage or restriction fragment length polymorphism (RFLP) due to its immunological significance and polymorphic characters. In some previous studies, the GP5 exhibited extensive variations among different PRRSV field strains, which might have important implications in immunology, being responsible for the limited cross protection [33, 34]. Therefore, it is valuable to monitor the variation of PRRSV GP5 and analyze its characteristics. It is shown that the ORF5 gene of CHbj2101 shared 84.2%–91.9% of nucleotide identity with the reference strains VR2332, CH1a, JXA1, JXA1 P80, NADC30, CHsx1401, NADC34, LNWK130, QYYZ, and 15LN3, respectively. Correspondingly, the amino acid identities of these representative strains were 83.6%–96.0%, sharing the highest similarity with the NADC30 virus (Table S2). The sequence alignment for the other two isolates was also summarized in Table S3 (for CHbj2102) and Table S4 (for CHbj2103).

**Figure 3:**
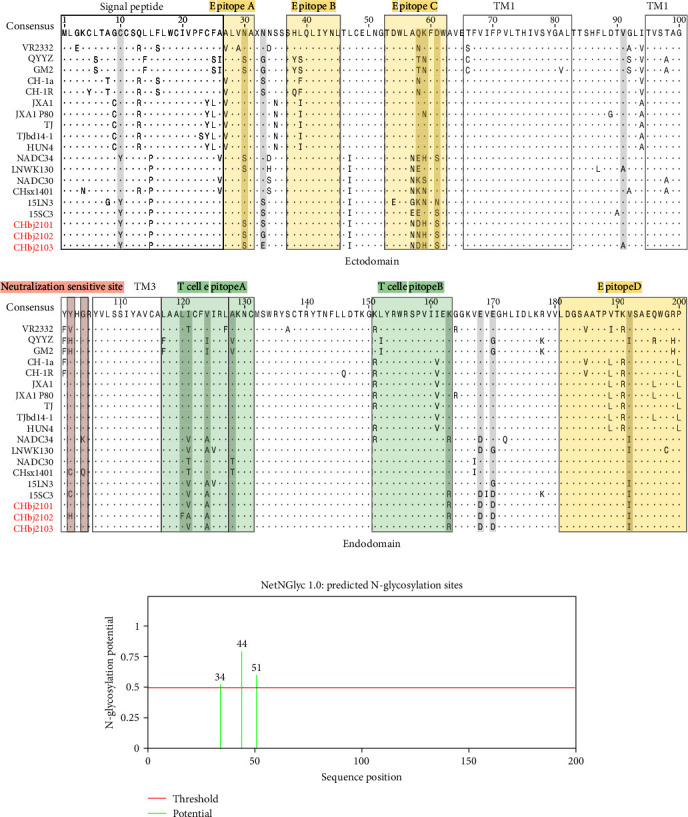
Alignment and comparison of amino acid mutations of GP5. (a)The signal peptide and transmembrane (TM) regions were shown in black boxes, respectively. The linear antigenic epitopes, cellular epitopes, and positively sensitive sites were indicated in yellow, green, and red, respectively. The characteristic amino acids of the three isolates were shown in gray. (b)Three predicted N-glycosylation sites of GP5 protein were shown at the bottom.

In the previous studies, the immune characteristics of GP5 have been widely explored [35,^36^]. GP5 is enriched with abundant antigenic epitopes that can induce antibodies in the infected pigs and is involved in stimulating the secretion of IFN-*γ* [37]. Epitope A, located at the position of aa 27–31, has been identified as a decoy epitope, inducing nonneutralizing antibodies against PRRSV GP5. The epitope B at the position aa 37–45, working as a primary neutralizing epitope (PNE) is conserved among the field strains, but it is not immunodominant. As well, epitope C and epitope D were involved in homologous neutralizing. Meanwhile, the amino acid diversity can be also found in the region of two identified *T*-cell epitopes (namely, aa 117–131 and aa 151–163). Among these epitopes, the main antigen recognition sites are characterized by residues H38, I42, Y43, and N44, while residues L39, Q40, and L41 are specifically involved in antibody binding. [38, 39]. Besides, the residues 102 and 104 of GP5 were also reported to determine the viral susceptibility to porcine serum neutralization, residue 102 was variable and 104 was highly conserved [40]. It could be noted that the degrees of difference between the new isolates and NADC30/NADC30-like PRRSV were varying in this study (Figure 3(a)). Ten collective amino acid substitutions, including C10Y, N30S, N59H, D61S, V124A, T128A, K163R, E168D, E170D, and V192I, were compared with the representative strains. Interestingly, seven of them had high homology with NADC34 or NADC34-like PRRSV. At the same time, there was one characteristic mutation in both CHbj2101 and CHbj2103 at epitope C (K58D), as well as two characteristic mutations in the *T* cell epitope A (L120F and I121A) of CHbj2102, were unique, when compared to other PRRSV strains. As expected, mutations rarely occurred in the signal peptide coding region (aa 1–31) and transmembrane (TM) domain (aa 65–82, aa 95–103, and aa 106–128).

As the glycosylation of GP5 is thought to be closely associated with helping viruses to escape the immune response and persist by effectively masking neutralizing antibodies, the variation in potential N-glycosylation sites (NGSs) was also predicted by using the NetNGlyc 1.0 server, to acquire more insight into the genetic evolution of these three isolates. GP5 of PRRSV-2 contains 3–5 potential NGSs, among these sites, N44 and N51 are conserved, meanwhile, the sites preceding N44 exhibit considerable variability and are vital for the PRRSV to evade antibody neutralization. In this study, all three isolates share the conserved pattern of N34, N44, and N51, just like the strains NADC30 and QYYZ (Figure 3(b)).

### 3.5. Sequence Analysis of Minor Glycoproteins

**Figure 4:**
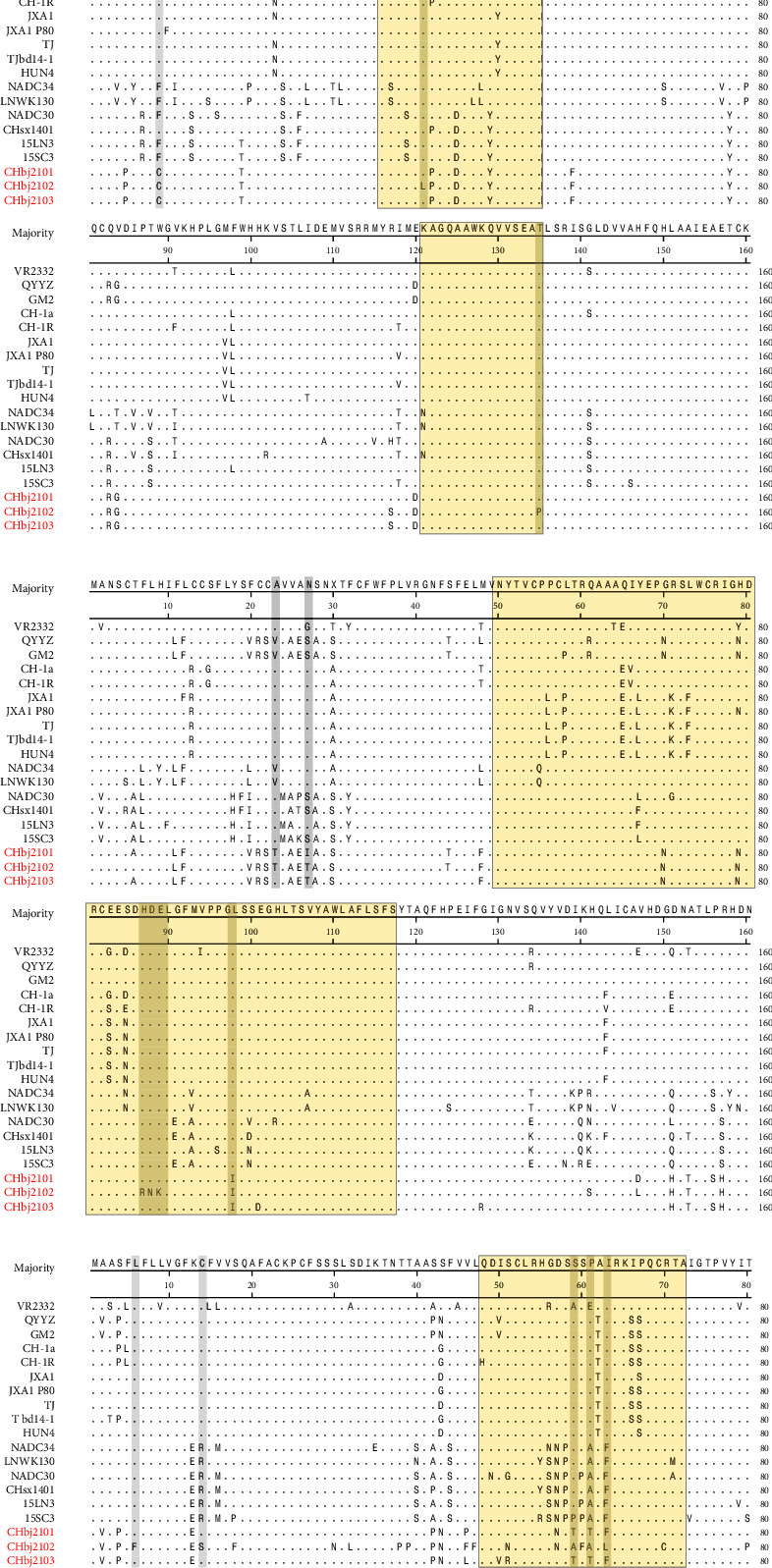
Alignment and comparison of amino acid mutations of GP2 (a), GP3 (b), and GP4 (c). The linear antigenic epitopes and positively sensitive sites were indicated in yellow and red, respectively. The amino acids characteristic of the three isolates were shown in gray.

The GP2-4 of these three isolates was similar to that of QYYZ. Two linear B-cell epitopes have been previously identified at aa 36–55 and aa 121–135 of GP2 [41] and the sequence alignment indicated that both CHbj2101 and CHbj2103 were conserved in these two regions. But for CHbj2102, two positively selected sites were observed at positions aa 35 and aa 41, respectively (Figure 4(a)). For PRRSV-2, there are four consecutively overlapping B-cell epitopes in GP3, located in aa 50–117 [42]. All these three isolates were highly variable at position 108 within the B-cell epitope (Figure 4(b)). Another noticeable point was that two novel mutation sites emerged at positions 59 and 61 within the GP4 B-cell epitope of CHbj2101 or CHbj2103 (Figure 4(c)). Except for these mutations in the epitopes, four positively selected sites were also observed in other regions, including aa 9 of GP2, aa 23 and aa 27 of GP3, and aa 6 and aa 14 of GP4. Considering the receptor binding function of these minor glycoproteins, these mutated sites might contribute to the viruses' immune escape, infection efficiency, or survival in the host population.

### 3.6. Phylogenetic Analysis

**Figure 5:**
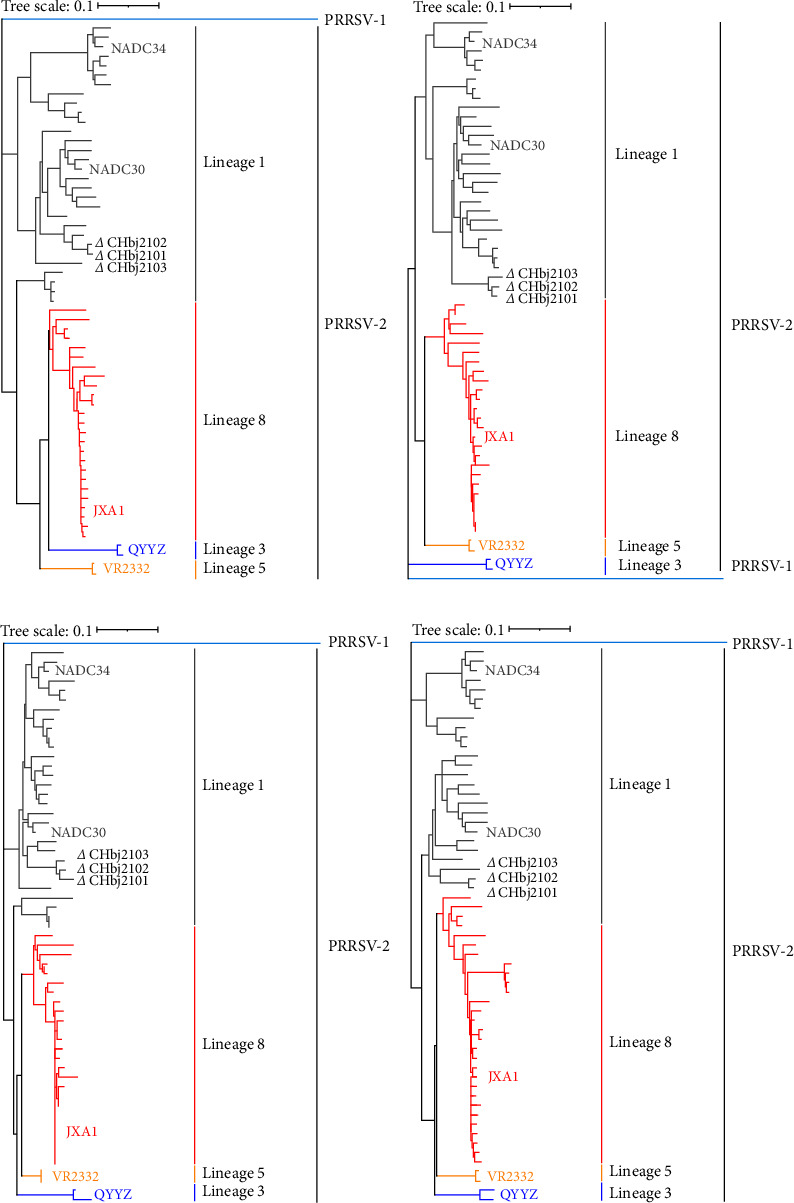
Phylogenetic trees of three strains isolated in this study based on the nucleotide sequences of nsp2 (a), ORF5 (b), ORF7 (c), and complete genome (d). Lineages 1, 3, 5, and 8 were highlighted in gray, blue, pink, and red, respectively. The representative strains in lineages were labeled with different colors and the three strains isolated in this study were labeled with triangles (△).

To further identify the evolutionary relations of these three isolates with representative strains from different lineages, phylogenetic tree based on the sequences of nsp2, ORF5, ORF7, and full-length genomes were carried out. As shown in Figure 5, these three isolates are clustered into the same branches in all four phylogenetic trees, which are all close to the reference strain NADC30 from Lineage 1. Moreover, CHbj2101 and CHbj2102 were closer to each other, when compared with CHbj2103.

### 3.7. Recombination Analysis

PRRSV genomic recombination is frequently observed in the field, which has been regarded as one of the most important strategies to drive PRRSV evolution. Recombination events in interlineage and intralineage both promote the emergence of novel epidemic isolates [43]. Since the introduction of NADC30-like virus in China, the field strains with recombination have been increasingly isolated [44]. It is noticed that the sequence homology of these three isolates to different representative strains varied in different coding regions. As shown in Tables S2–S4, most coding regions of these three isolates shared higher nucleotide/amino acid identity with NADC30 and Chinese NADC30-like strain CHsx1401, but the region of 5′UTR, nsp1*α*, nsp1*β*, nsp4-nsp9 (for CHbj2101 and CHbj2102) exhibited higher identity with JXA1 P80, a HP-PRRSV derived MLV strain. In addition, GP2-4 of the three isolates showed the highest identity with QYYZ. These results suggested that they might be recombinant viruses.

**Figure 6:**
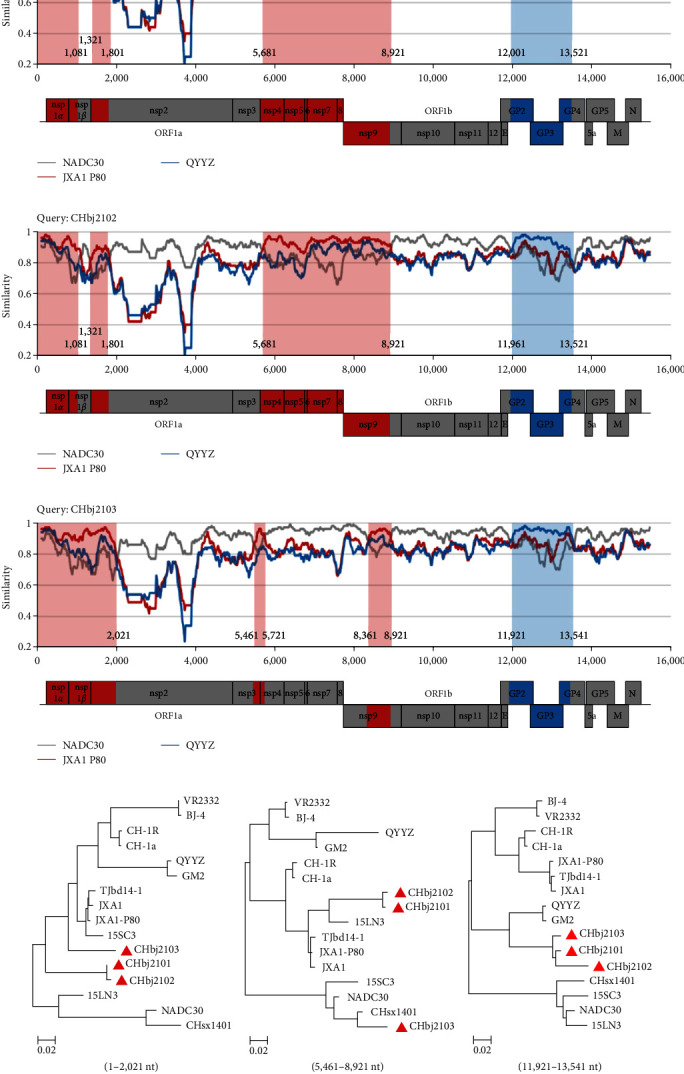
Genome recombination analysis of the strains CHbj2101 (a), CHbj2102 (b), and CHbj2103 (c). Recombination analysis was calculated by Simplot v.3.5.1 software. The genome-scale similarity was the comparison of the isolates (query) with NADC30 (gray), JXA1 P80 (red), and QYYZ (blue). The major parental regions were in gray, whereas the minor parental regions were in red and blue, respectively. For Similarity plot analysis, the *y*-axis indicated the percentage identity in a sliding 200 bp window with 20 bp steps. (d–f) The phylogenic trees based on the recombinant regions were constructed. Three isolated strains were labeled with red triangles (△).

To determine the recombination events, the full-length genomic sequences of these three isolates were examined by RDP4 and confirmed by SimPlot v.3.5.1 with the sequences of strains NADC30, JXA1 P80, and QYYZ as references. The results suggested that CHbj2101, CHbj2102, and CHbj2103 were all identified as recombinant strains among strains NADC30, JXA1 P80, and QYYZ (Figure 6). The isolates CHbj2101 and CHbj2102 shared the same recombination pattern. For the isolate CHbj2101, it possesses seven recombination breakpoints distributed in nsp1*β* (nt 1,081 and nt 1,321), nsp2 (nt 1,801), nsp4 (nt 5,681), nsp9 (nt 8,921), ORF2a (nt 12,001), and ORF4 (nt 13,521), separating the genome of CHbj2101 into eight regions (Figure 6(a)). CHbj2103 presents a similar recombinant pattern in the structural protein coding region, but for the nonstructural protein coding regions, the recombination pattern is different, with breakpoints at nsp2 (nt 2,021), nsp3 (nt 5,461), nsp4 (nt 5,721), and nsp9 (nt 8,361) (Figure 6(c)). The additional phylogenetic analysis on the recombinant regions (Figure 6(d)–6(f)) can further confirm the interlineage recombination.

### 3.8. Interlineage Recombination Analysis of QYYZ-Like Strains

**Figure 7:**
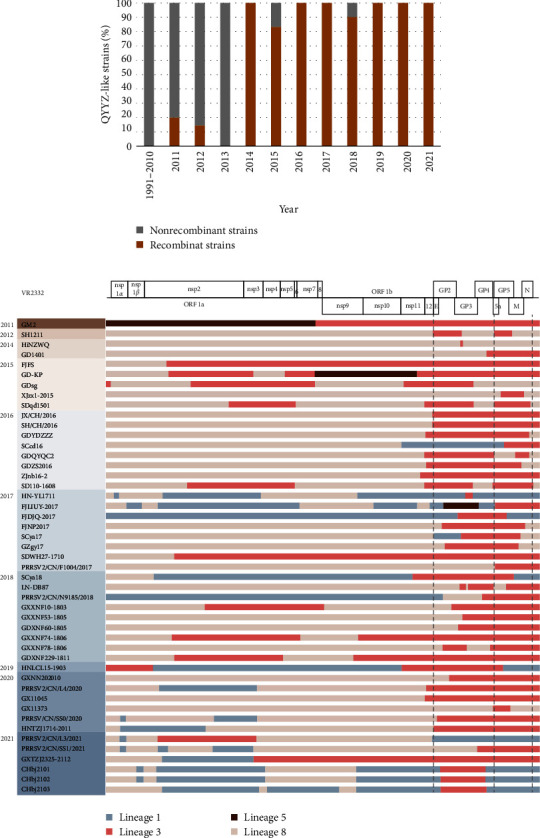
Recombination analysis of QYYZ-like PRRSV strains reported in China from the year 1991–2021. (a) Annual percentage of QYYZ-like strains in China. Gray indicated nonrecombinant QYYZ-like strains (*n* = 36) and tangerine indicated interlineage recombinant QYYZ-like strains (*n* = 47). (b) Interlineage recombination hot regions analysis of QYYZ-like PRRSV strains. Different colors indicated different lineages. The positions were according to the VR2332 strain.

Considering the NADC30-like virus has become a predominant epidemic in China, once QYYZ-like PRRSV was inducted into the pig farm, there will be a high possibility to cause a new outbreak in the PRRS stable herd, leading to highly frequent recombination among different strains. Therefore, it is necessary to increase the awareness of QYYZ-like PRRSV strains monitoring in the field continuously. To further investigate the recombination characteristics of the QYYZ-like virus in China, we collected all currently available genomic sequences for recombination analysis (Table S5). The recombination analysis was performed by using nine representative parental strains from different lineages of PRRSV-2 as references. Among these QYYZ-like viruses reported between 1991 and 2021 in China, 47 of 83 strains were identified as interlineage recombinant viruses. Especially, the percentage of recombinant strains explosively increased over the past 10 years (Figure 7(a)). By consolidating the recombination data from the previous study, recombination events have been detected since 2011, and the dominant parent strains were from Lineage 8 PRRSV during the years 2012–2015, which was possibly related to the widespread use of the HP-PRRSV-derived MLV [45, 46]. After the year 2015, the dominant parent strains have been replaced by Lineage 1 NADC30-like virus in the field [47, 48]. Among these recombinant events, the Lineage 8 and Lineage 1 viruses played the role of major parent strains, except for only a few recombinants with the Lineage 3 virus as the major parent strain. For recombinant viruses, QYYZ-like strains were the mainly fragments provider, supplying the structural protein-coding regions (especially GP2-GP4). Meanwhile, nsp2–nsp7 was also a recombination hot region (12/47, 25.53%) beyond our expectations (Figure 7(b)).

In general, recombinant breakpoints were observed at various positions spanning the entire genome of PRRSV. In previous studies, the hot spots of the high-frequency interlineage recombination, mainly between Lineage 1 and Lineage 8, were often identified into nsp1, nsp4, nsp7, nsp9, nsp12, ORF2, and ORF3. However, the recombination breakpoints of the QYYZ-like virus were dispersed and exhibited a complex distribution pattern throughout the genome. Many breakpoints were concentrated in the nsp2, nsp7, nsp11, ORF2, ORF4, and ORF7 (Table S6).

## 4. Discussion

The genetic diversity and frequent emergence of novel variants greatly hinder the prevention and control efforts of PRRSV in the last 30 years. Different kinds of strains with great diversity are concomitantly circulating in the field. Currently, the main pandemic virus in China can be further classified into four lineages of PRRSV-2, including Lineage 1 (sublineage 1.8 NADC30-like virus and sublineage 1.5 NADC34-like virus), Lineage 3 (QYYZ-like virus), Lineage 5 (VR2332-like virus), and Lineage 8 (CH-1a-like virus/HP-PRRSV). The Lineage 1 strains are currently predominant viruses in China [49], which are also epidemic in North America [50, 51] and other Asian countries [44]. Additionally, several previous studies have indicated that the NADC30-like virus shows a high propensity of frequent recombination with other lineage strains, encompassing both field viruses and vaccine strains [49, 52, 53]. For the Lineage 3 virus, its prototypic strain MD001 was first found in the Taiwan province in the early 1990s [54]. Whereafter, Lineage 3 PRRSV appeared in Hong Kong (2004) and mainland China (2005) [46]. QYYZ, the representative isolate of Lineage 3 virus, was initially detected in mainland China in the year 2010, then gradually gained prevalence in southern China and exhibited an expanding genetic diversity [55, 56]. After three decades of transmission and evolution, QYYZ-like strains have become widespread in mainland China, Hong Kong, and Taiwan area. Early studies have shown that QYYZ-like strains were sporadically reported in 2015 and subsequently rapidly disseminated throughout southern China [31, 56, 57]. At the same time, the discovery of a QYYZ and MLV recombinant strain GM2 in Guangdong Province, has sparked extensive interest in QYYZ-like strains [58]. It is noticed that all newly found QYYZ-like strains in mainland China were recombinant strains, and the nonrecombinant original QYYZ strain has not been found to circulate in the field yet [59].

In this study, three novel PRRSV isolates named CHbj2101, CHbj2102, and CHbj2103 were successfully isolated from tissue samples collected from three different nursery barns in the same pig farm. The sequence analysis demonstrated that these three isolates all belonged to Lineage 1 PRRSV. Even though they are different strains with varied site mutations and different recombination patterns, these three strains still share high sequence homology, especially among their nonrecombinant backbone regions. The samples with CHbj2101 or CHbj2102 were collected at the early stage of this outbreak, with weeks intervals, meanwhile, the samples with CHbj2103 and samples that the virus has not been successfully isolated from were collected at the end of this outbreak. It is speculated that these viruses might be introduced into the farm from a single event, and then, they are under different evolutionary processes. After competition and adaptation in the herd, one strain might become the predominant virus on this farm.

Mutagenesis is an important mechanism driving PRRSV evolution, which facilitates the virus in adapting to its host or evading the immune response, leading to the emergence of a more highly adapted virus. This kind of virus tends to become the dominant strain in the quasispecies swarm. GP2, GP3, and GP4 form a trimeric complex in order to interact with the receptor CD163 and mediate PRRSV infection, and the GP5 is also essential for virion formation [60–63]. Meanwhile, GP5 is the most variable one among these structural proteins [14]. Compared the GP5 coding sequences of these three isolates with that of representative strains, they share almost 70% of the collective amino acid substitutions with that in NADC34 or NADC34-like PRRSV, but the mutagenesis sites among these three isolates were varied. At the same time, GP5 also possesses multiple NGSs. Among these, N44 and N51 are highly conserved glycosylation sites across various PRRSV strains, while NGSs at other sites tend to be specific to particular strains [64]. In this study, N34, N44, and N51 can be observed in GP5. Curiously, compared with NGSs in other NADC30-like strains, the pattern of these three NGSs seems to be less found. Considering some of these mutations might be able to alter the antigenicity of proteins, benefiting viral immune evasion, an additional research should be required to explore the effects of this NGS pattern.

Recombination has been also recognized as a significant factor contributing to the diversity of PRRSV [46, 65]. The recombinants in these three isolates evolved in a complex pattern (interlineages) of Lineages 1 + 8 + 3. It is noteworthy that the recombination patterns of the three isolates were exactly similar to 15LN3 or 15SC3 except for GP2–GP4. A previous study has shown that the 15LN3 was a recombinant virus with parental strains NADC30, HP-PRRSV, and JXA1-R (JXA1 P80). And 15SC3 was based on NADC30 as the parent strain, with JXA1-R providing recombinant fragments. These strains may provide the fragments for recombination. In previous studies, it is shown that most of the hot recombination regions were identified in nsp2 or nsp9 coding regions between NADC30-like viruses and other PRRSV strains in China [66, 67]. In the present study, the hot regions for breakpoint were identified in nsp1, nsp2, nsp4, nsp9, GP2, and GP3, indicating that nsp2 or nsp9 were still the hot recombination regions in the nonstructural protein. The latest result in our laboratory indicated that nsp2 was a critical virulence regulator and an inducer of host immune responses, especially in the part of inflammation. Recombinant nsp2 region from NADC30-like virus might benefit from reducing the activation of the inflammatory response and increasing the viral persistence via immune modulation [68]. GP2 and GP4 are regarded as the main viral proteins that interact with the CD163 receptor, contributing to PRRSV entry, and the GP2–GP4 of the three isolates showed higher identity with QYYZ, which might affect the antigenic characteristics of the virus. The recombination in these regions is thought to be linked with enhancing proliferation capacity and cellular tropism of PRRSV, which contribute to the survival, spread, adaptability, and ultimately influence the pathogenesis. Whether these events are associated with virulence changes and immune escape still needs further study in the future.

## 5. Conclusion

In summary, three novel recombinant PRRSV strains, CHbj2101, CHbj2102, and CHbj2103 are successfully isolated, which were the recombinants from Lineages 1, 8, and 3. The major parental strain was the NADC30-like virus and contained fragments from HP-PRRSV vaccine-like strains and QYYZ-like strains. The findings in this study have valuable implications for PRRSV recombination dynamics and emphasize the importance of inspection on pig farms to reduce the likelihood of novel recombinant variants emerging and causing new outbreaks. These findings further underscore the significance of the study on PRRSV pathogenicity, immunogenicity, and diagnostic methods, providing more comprehensive knowledge on the potential significance of evolution and strengthening control measures of PRRSV.

## Figures and Tables

**Table 1:** The whole-genomic nucleotide identity among the three isolates and PRRSV reference strains (%).

Strain	CHbj2101	CHbj2102	CHbj2103
CHbj2101	/	/	/
CHbj2102	98.8	/	/
CHbj2103	91.1	90.9	/
VR2332	85.2	85.1	85.2
CH-1a	85.9	85.8	85.4
JXA1	86.3	86.1	85.6
JXA1 P80	86.2	86	85.5
NADC30	87.5	87.3	88.8
CHsx1401	87.4	87.3	89.8
NADC34	84.2	84	84.5
LNWK130	83.7	83.6	83.9
QYYZ	84.1	84	83.8
15LN3	90.1	89.9	88.6
15SC3	88.6	88.5	90.8

## Data Availability

The data used to support the findings of this study are included within the article and in the GenBank database.

## Supplementary Materials

Supplementary Materials Table S1: information of the PRRSV reference strains used for phylogenetic analysis in this study. Table S2: nucleotide and amino acid identities of different regions of CHbj2101 genome with other representative PRRSV strains. Table S3: nucleotide and amino acid identities of different regions of CHbj2102 genome with other representative PRRSV strains. Table S4: nucleotide and amino acid identities of different regions of CHbj2103 genome with other representative PRRSV strains. Table S5: information on the whole genomes of all reported QYYZ-like PRRSV strains in China from 1991 to 2021. Table S6: recombination information of QYYZ-like PRRSV in 1991–2021.
