# Supplementary material for: Genetic Characteristics of Three Single-Farm-Isolated Porcine Reproductive and Respiratory Syndrome Viruses with Novel Recombination among NADC30-Like, JXA1-Like, and QYYZ-Like Strains
Source: Transbound Emerg Dis. 2023 Jul 22;2023:8871321. doi: 10.1155/2023/8871321 (PMC12017160; doi:10.1155/2023/8871321)
Supplement: Supplementary Materials — Table S1: information of the PRRSV reference strains used for phylogenetic analysis in this study. Table S2: nucleotide and amino acid identities of different regions of CHbj2101 genome with other representative PRRSV strains. Table S3: nucleotide and amino acid identities of different regions of CHbj2102 genome with other representative PRRSV strains. Table S4: nucleotide and amino acid identities of different regions of CHbj2103 genome with other representative PRRSV strains. Table S5: information on the whole genomes of all reported QYYZ-like PRRSV strains in China from 1991 to 2021. Table S6: recombination information of QYYZ-like PRRSV in 1991–2021. [file 8871321.f1.docx]

**Supplementary Table 1** Information of the PRRSV reference strains used for phylogenetic analysis in this study

| **No.** | **Strain** | **Accession no.** | **Year** | **Country** | **Province**  **/States** | **Type** | **Lineage** |
| --- | --- | --- | --- | --- | --- | --- | --- |
| 1 | Lelystad virus | M96262 | 1993 | Netherlands | NA^a^ | 1 |  |
| 2 | MN184A | DQ176019 | 2006 | USA | Minnesota | 2 | 1 |
| 3 | MN184B | DQ176020 | 2006 | USA | Minnesota | 2 | 1 |
| 4 | MN184C | EF488739 | 2007 | USA | NA^a^ | 2 | 1 |
| 5 | NADC30 | JN654459 | 2008 | USA | Iowa | 2 | 1 |
| 6 | NADC34 | MF326985 | 2014 | USA | Iowa | 2 | 1 |
| 7 | XW015 | KF724409 | 2013 | USA | NA^a^ | 2 | 1 |
| 8 | CHsx1401 | KP861625 | 2014 | China | Shanxi | 2 | 1 |
| 9 | HeN1401 | MF766471 | 2014 | China | Henan | 2 | 1 |
| 10 | 15HEN1 | KX815413 | 2015 | China | Henan | 2 | 1 |
| 11 | 15HEN4 | KX815415 | 2015 | China | Henan | 2 | 1 |
| 12 | SC-d | MF375261 | 2015 | China | Sichuan | 2 | 1 |
| 13 | HNhx | KX766379 | 2016 | China | Henan | 2 | 1 |
| 14 | HENXX-8 | KY041782 | 2016 | China | Henan | 2 | 1 |
| 15 | HeN1601 | MF766474 | 2016 | China | Henan | 2 | 1 |
| 16 | SX1-1607 | MN046240 | 2016 | China | Shanxi | 2 | 1 |
| 17 | SX2-1607 | MN046241 | 2016 | China | Shanxi | 2 | 1 |
| 18 | HeB-239 | MN046229 | 2018 | China | Hebei | 2 | 1 |
| 19 | HLJWK108-1711 | MN046230 | 2018 | China | Heilongjiang | 2 | 1 |
| 20 | LNWK96 | MG860516 | 2017 | China | Liaoning | 2 | 1 |
| 21 | LNWK130 | MG913987 | 2017 | China | Liaoning | 2 | 1 |
| 22 | LNDZD10-1806 | MN648054 | 2018 | China | Liaoning | 2 | 1 |
| 23 | HLJZD22-1812 | MN648450 | 2018 | China | Heilongjiang | 2 | 1 |
| 24 | FJ0908 | MK202794 | 2018 | China | Fujian | 2 | 1 |
| 25 | CH/2018/NCV-Anheal-1 | MH370474 | 2018 | China | NA^a^ | 2 | 1 |
| 26 | HLJZD30-1902 | MN648055 | 2019 | China | Heilongjiang | 2 | 1 |
| 27 | QYYZ | JQ308798 | 2010 | China | Guangdong | 2 | 3 |
| 28 | GM2 | JN662424 | 2011 | China | Guangdong | 2 | 3 |
| 29 | VR2332 | [U87392](https://www.ncbi.nlm.nih.gov/nuccore/U87392) | 1992 | USA | NA^a^ | 2 | 5 |
| 30 | BJ-4 | AF331831 | 1996 | China | Beijing | 2 | 5 |
| 31 | P129 | MK820651 | 1995 | USA | Indiana | 2 | 8 |
| 32 | CH-1a | AY032626 | 1996 | China | Beijing | 2 | 8 |
| 33 | HB-1(sh)/2002 | AY150312 | 2001 | China | Hebei | 2 | 8 |
| 34 | HB-2(sh)/2002 | AY262352 | 2001 | China | Hebei | 2 | 8 |
| 35 | JXA1 | EF112445 | 2006 | China | Jiangxi | 2 | 8 |
| 36 | HUN4 | EF635006 | 2006 | China | Hunan | 2 | 8 |
| 37 | TJ | EU860248 | 2006 | China | Tianjin | 2 | 8 |
| 38 | JXA1 P80 | FJ548853 | 2006 | China | Beijing | 2 | 8 |
| 39 | BJ0706 | GQ351601 | 2007 | China | Beijing | 2 | 8 |
| 40 | Em2007 | EU262603 | 2007 | China | Hubei | 2 | 8 |
| 41 | Henan-1 | EU200962 | 2007 | China | Henan | 2 | 8 |
| 42 | 07HEN | FJ393457 | 2007 | China | Henan | 2 | 8 |
| 43 | JXwn06 | EF641008 | 2008 | China | Jiangxi | 2 | 8 |
| 44 | CH-1R | EU807840 | 2008 | China | Heilongjiang | 2 | 8 |
| 45 | HLJ-09 | HQ843178 | 2009 | China | Henan | 2 | 8 |
| 46 | HLM-09 | HQ843179 | 2009 | China | Henan | 2 | 8 |
| 47 | HeN1201 | MF689000 | 2012 | China | Henan | 2 | 8 |
| 48 | HeN1301 | MF766470 | 2013 | China | Henan | 2 | 8 |
| 49 | HeN1501 | MF766472 | 2015 | China | Henan | 2 | 8 |
| 50 | HeN1502 | MF766473 | 2015 | China | Henan | 2 | 8 |
| 51 | TJbd14-1 | KP742986 | 2014 | China | Tianjin | 2 | 8 |
| 52 | 15HEN3 | KX815414 | 2015 | China | Henan | 2 | 8 |
| 53 | 15HUN1 | KX815416 | 2015 | China | Henan | 2 | 8 |
| 54 | 15HUN2 | KX815417 | 2015 | China | Henan | 2 | 8 |
| 55 | 15HUN3 | KX815418 | 2015 | China | Henan | 2 | 8 |
| 56 | 15LN3 | KX815425 | 2015 | China | Liaoning | 2 | 1 |
| 57 | 15SC3 | KX815428 | 2015 | China | Sichuan | 2 | 1 |
| 58 | CHbj2101^b^ | OP734316 | 2021 | China | Beijing | 2 | 1 |
| 59 | CHbj2102^b^ | OP734317 | 2021 | China | Beijing | 2 | 1 |
| 60 | CHbj2103^b^ | OP734318 | 2021 | China | Beijing | 2 | 1 |

^a^ Not available.

^b^ Strains isolated in this study.

**Supplementary Table 2** Nucleotide and amino acid identities of different regions of CHbj2101 genome with other representative PRRSV strains

| **Region** | **Pairwise % Identity (nt/aa)** | | | | | | | | | | | |
| --- | --- | --- | --- | --- | --- | --- | --- | --- | --- | --- | --- | --- |
|  | **VR2332** | **CH-1a** | **JXA1** | **JXA1 P80** | **NADC30** | **CHsx1401** | **NADC34** | **LNWK130** | **QYYZ** | **15LN3** | **CHbj2102** | **CHbj2103** |
| 5‘UTR | 90.3 | 95.7 | 96.8 | 96.3 | 89.8 | 89.3 | 91.9 | 90.3 | 94.7 | 96.3 | 100 | 99.5 |
|  | / | / | / | / | / | / | / | / | / | / | / | / |
| Nsp1α | 89.1 | 92.8 | 92.8 | 93.3 | 88 | 86.7 | 86.7 | 88.1 | 91.7 | 92.6 | 99.8 | 97 |
|  | 95 | 96.1 | 98.3 | 98.3 | 95 | 93.9 | 95.6 | 96.7 | 96.1 | 98.3 | 99.4 | 98.3 |
| Nsp1β | 83.4 | 84.9 | 87.8 | 87.2 | 81.9 | 80.6 | 77.8 | 76.7 | 80.5 | 82.6 | 99.8 | 88 |
|  | 78.3 | 79.8 | 85.7 | 85.2 | 78.3 | 74.9 | 70.4 | 69.5 | 76.8 | 77.3 | 99.5 | 88.7 |
| Nsp2 | 79 | 77.7 | 77.6 | 76 | 88.6 | 86.4 | 77.1 | 76.4 | 74.7 | 87.1 | 99.1 | 84.8 |
|  | 73.9 | 72.4 | 72.6 | 71.8 | 85.1 | 83.5 | 72.5 | 72.2 | 68.6 | 83 | 99.2 | 82.2 |
| Nsp3 | 83.8 | 83.6 | 82.2 | 82.3 | 89.4 | 91.2 | 84.6 | 83.9 | 80.9 | 90.3 | 98.4 | 87.2 |
|  | 89.1 | 87.4 | 87.4 | 87.4 | 91.3 | 93 | 89.1 | 88.3 | 87 | 94.3 | 99.6 | 93 |
| Nsp4 | 87.7 | 91.5 | 94.8 | 94.8 | 81.5 | 81.5 | 84 | 84.2 | 83 | 92.8 | 98.9 | 81.7 |
|  | 91.2 | 92.2 | 95.1 | 95.1 | 91.2 | 91.7 | 91.7 | 91.2 | 90.2 | 95.1 | 99 | 91.7 |
| Nsp5 | 83.7 | 87.6 | 89.6 | 90 | 80.8 | 81.2 | 80.6 | 80.2 | 79 | 88.6 | 98.2 | 81.2 |
|  | 87.6 | 92.4 | 92.9 | 92.9 | 87.6 | 87.1 | 84.7 | 84.1 | 86.5 | 90 | 98.2 | 87.1 |
| Nsp6 | 93.8 | 95.8 | 97.9 | 97.9 | 85.4 | 89.6 | 54.2 | 81.2 | 95.8 | 95.8 | 97.9 | 81.2 |
|  | 93.8 | 100 | 100 | 100 | 100 | 87.5 | 87.5 | 100 | 100 | 100 | 100 | 87.5 |
| Nsp7 | 86.6 | 92.8 | 95 | 95.2 | 80.3 | 80.3 | 79.8 | 79.8 | 90.6 | 93.4 | 99.2 | 80.1 |
|  | 88 | 93.1 | 95 | 95.4 | 82.6 | 83.4 | 84.9 | 85.3 | 91.9 | 93.1 | 99.2 | 83.4 |
| Nsp8 | 92 | 93.5 | 92.8 | 93.5 | 87 | 84.8 | 84.8 | 85.5 | 88.4 | 93.5 | 99.3 | 84.8 |
|  | 95.7 | 95.7 | 95.7 | 95.7 | 89.1 | 88.9 | 89.1 | 87 | 95.7 | 95.7 | 100 | 89.1 |
| Nsp9 | 88.3 | 89.7 | 90.5 | 90.4 | 89.3 | 89.6 | 87.5 | 87 | 87.2 | 93.8 | 99.3 | 94.4 |
|  | 97 | 97.2 | 97.5 | 97.8 | 96.2 | 96.4 | 96.7 | 96 | 96 | 97.8 | 99.7 | 97.2 |
| Nsp10 | 85 | 84.9 | 84.4 | 84.4 | 92.1 | 92.7 | 89.9 | 90.2 | 84 | 92.7 | 99.3 | 96.4 |
|  | 95.2 | 94.1 | 94.6 | 94.8 | 97.5 | 98.6 | 98.2 | 98.4 | 94.6 | 97.7 | 100 | 96.8 |
| Nsp11 | 87.9 | 88.6 | 88 | 87.6 | 91.3 | 88.5 | 84.6 | 84.6 | 85.1 | 91 | 98.5 | 91.9 |
|  | 94.6 | 96.4 | 97.3 | 96.4 | 96 | 94.2 | 94.6 | 94.2 | 93.7 | 93.7 | 98.7 | 97.3 |
| Nsp12 | 88.3 | 87.4 | 86.4 | 86.4 | 88.1 | 88.3 | 85.1 | 85.3 | 89.8 | 89.4 | 99.1 | 97.2 |
|  | 95.5 | 96.1 | 94.8 | 94.8 | 94.2 | 93.5 | 92.9 | 92.9 | 96.8 | 92.9 | 99.4 | 98.7 |
| ORF2a | 89.8 | 89.4 | 89.4 | 89.4 | 84.2 | 86 | 85.6 | 85.1 | 96.4 | 85.6 | 98.3 | 98.8 |
|  | 90.6 | 90.3 | 89.1 | 89.1 | 85.9 | 89.1 | 84.8 | 83.3 | 97.7 | 87.9 | 96.9 | 98.4 |
| ORF2b | 93.7 | 93.2 | 92.8 | 93.2 | 89.6 | 91.4 | 87.8 | 86.5 | 97.7 | 91.4 | 100 | 99.1 |
|  | 94.5 | 89.2 | 90.5 | 90.5 | 91.9 | 90.5 | 93.2 | 98.6 | 90.5 | 90.5 | 100 | 100 |
| ORF3 | 86 | 87.5 | 87.7 | 87.2 | 81.6 | 83 | 83.1 | 81.8 | 94.2 | 83.1 | 94.4 | 98.2 |
|  | 80.7 | 83.5 | 83.9 | 83.9 | 79.6 | 80.8 | 79.2 | 76.9 | 92.2 | 80.8 | 91 | 97.3 |
| ORF4 | 86.6 | 87.9 | 86.4 | 87.3 | 90.1 | 90.1 | 91.6 | 90.3 | 88.1 | 91.2 | 92.6 | 98.7 |
|  | 87.1 | 89.9 | 88.8 | 89.9 | 87.7 | 89.9 | 90.5 | 88.8 | 90.5 | 88.8 | 84.9 | 97.2 |
| ORF5a | 85.8 | 87.9 | 85.1 | 82.3 | 94.2 | 94.3 | 92.2 | 90.8 | 86.5 | 92.2 | 99.3 | 97.2 |
|  | 85.1 | 87.2 | 83 | 83 | 91.3 | 91.5 | 95.7 | 93.6 | 80.9 | 89.4 | 97.9 | 93.6 |
| ORF5 | 84.2 | 86.2 | 84.7 | 84.6 | 91.5 | 91.9 | 87.2 | 86.6 | 83.3 | 90.4 | 98.8 | 97.5 |
|  | 83.6 | 86.1 | 85.6 | 84.6 | 96 | 93.5 | 91 | 88.6 | 84.1 | 94 | 97.5 | 99 |
| ORF6 | 89.9 | 87.2 | 88.6 | 88.2 | 94.9 | 93.7 | 92.6 | 92.4 | 88.4 | 95 | 99.8 | 98.1 |
|  | 93.1 | 90.9 | 92 | 91.4 | 98.3 | 96.6 | 93.1 | 94.3 | 92 | 98.3 | 100 | 96.6 |
| ORF7 | 89 | 88.4 | 87.4 | 87.4 | 92.7 | 90.3 | 90.9 | 90.1 | 86 | 90.2 | 98.1 | 97 |
|  | 90.3 | 90.3 | 88.7 | 88.7 | 94.4 | 89.5 | 90.3 | 91.1 | 87.9 | 90.2 | 97.6 | 96.8 |
| 3‘UTR | 92.7 | 90.7 | 91.3 | 90 | 98 | 97.4 | 95.4 | 94.7 | 90.1 | 96 | 98 | 98 |
|  | / | / | / | / | / | / | / | / | / | / | / | / |

**Supplementary Table 3** Nucleotide and amino acid identities of different regions of CHbj2102 genome with other representative PRRSV strains

| **Region** | **Pairwise % Identity (nt/aa)** | | | | | | | | | | | | | | | | | | | | | | |
| --- | --- | --- | --- | --- | --- | --- | --- | --- | --- | --- | --- | --- | --- | --- | --- | --- | --- | --- | --- | --- | --- | --- | --- |
|  | **VR2332** | **CH-1a** | | **JXA1** | | **JXA1 P80** | **NADC30** | | **CHsx1401** | | **NADC34** | | **LNWK130** | | **QYYZ** | | | **15LN3** | | **CHbj2101** | **CHbj2103** | |  |
| 5‘UTR | 90.3 | | 95.7 | | 96.8 | 96.3 | | 89.8 | | 89.3 | | 91.9 | | 90.3 | | 94.7 | 96.3 | | 100 | | 99.5 |  |  |
|  | / | | / | | / | / | | / | | / | | / | | / | | / | / | | / | |  |  |  |
| Nsp1α | 88.9 | | 92.6 | | 92.6 | 93.1 | | 87.8 | | 86.5 | | 86.5 | | 88 | | 91.5 | 92.4 | | 99.8 | | 96.9 |  |  |
|  | 94.4 | | 95.6 | | 97.8 | 97.8 | | 94.4 | | 93.9 | | 95 | | 96.1 | | 95.6 | 97.8 | | 99.4 | | 97.8 |  |  |
| Nsp1β | 83.6 | | 84.7 | | 87.7 | 87 | | 81.8 | | 80.5 | | 77.7 | | 76.5 | | 80.3 | 82.4 | | 99.8 | | 87.8 |  |  |
|  | 78.3 | | 79.8 | | 85.7 | 84.7 | | 78.3 | | 74.9 | | 70.4 | | 69.5 | | 76.8 | 76.8 | | 99.5 | | 88.2 |  |  |
| Nsp2 | 78.9 | | 77.9 | | 77.8 | 76.1 | | 88.7 | | 86.4 | | 77 | | 76.3 | | 74.7 | 87.2 | | 99.1 | | 84.9 |  |  |
|  | 73.6 | | 72.5 | | 72.7 | 71.8 | | 85.1 | | 83.8 | | 72.5 | | 72.1 | | 68.9 | 83.2 | | 99.2 | | 82.2 |  |  |
| Nsp3 | 84.2 | | 83.5 | | 82 | 82.2 | | 89.9 | | 91.3 | | 84.8 | | 83.8 | | 80.1 | 90.4 | | 98.4 | | 87.1 |  |  |
|  | 88.7 | | 87 | | 87 | 87 | | 90.9 | | 92.6 | | 88.7 | | 87.8 | | 86.5 | 93.9 | | 99.6 | | 92.6 |  |  |
| Nsp4 | 88.4 | | 91.8 | | 94.8 | 94.8 | | 81.5 | | 81.9 | | 85 | | 84.8 | | 83 | 92.5 | | 98.9 | | 82 |  |  |
|  | 92.2 | | 93.1 | | 96.1 | 95.6 | | 92.2 | | 92.6 | | 92.6 | | 92.2 | | 90.7 | 95.1 | | 99 | | 92.6 |  |  |
| Nsp5 | 84.3 | | 88.2 | | 90.2 | 90.6 | | 81.8 | | 82.2 | | 81.6 | | 81.2 | | 80 | 89.2 | | 98.2 | | 82.2 |  |  |
|  | 87.6 | | 92.4 | | 92.9 | 92.9 | | 88.8 | | 88.2 | | 85.9 | | 85.3 | | 87.6 | 90 | | 98.2 | | 88.2 |  |  |
| Nsp6 | 95.8 | | 97.9 | | 100 | 100 | | 87.5 | | 91.7 | | 52.1 | | 83.3 | | 97.9 | 97.9 | | 97.9 | | 83.3 |  |  |
|  | 93.8 | | 100 | | 100 | 100 | | 100 | | 87.5 | | 87.5 | | 100 | | 100 | 100 | | 100 | | 87.5 |  |  |
| Nsp7 | 86.5 | | 92.7 | | 94.7 | 95 | | 79.9 | | 79.9 | | 79.4 | | 79.4 | | 90.5 | 93.2 | | 99.2 | | 79.7 |  |  |
|  | 88 | | 92.7 | | 94.6 | 95 | | 82.6 | | 83.4 | | 84.9 | | 85.3 | | 91.5 | 92.7 | | 99.2 | | 83.4 |  |  |
| Nsp8 | 91.3 | | 92.8 | | 92 | 92.8 | | 86.2 | | 85.5 | | 85.5 | | 84.8 | | 87.7 | 92.8 | | 99.3 | | 84.1 |  |  |
|  | 95.7 | | 95.7 | | 95.7 | 95.7 | | 89.1 | | 88.9 | | 89.1 | | 87 | | 95.7 | 95.7 | | 100 | | 89.1 |  |  |
| Nsp9 | 88.3 | | 89.5 | | 90.3 | 90.2 | | 89.2 | | 89.4 | | 87.4 | | 86.9 | | 87 | 93.6 | | 99.3 | | 94.3 |  |  |
|  | 96.9 | | 97.2 | | 97.5 | 97.8 | | 96.1 | | 96.2 | | 96.7 | | 96 | | 96 | 97.8 | | 99.7 | | 97 |  |  |
| Nsp10 | 84.7 | | 84.7 | | 84.4 | 84.4 | | 92.4 | | 92.8 | | 90.2 | | 90.2 | | 83.7 | 92.6 | | 99.3 | | 96.4 |  |  |
|  | 95.2 | | 94.1 | | 94.6 | 94.8 | | 97.5 | | 98.6 | | 98.2 | | 98.4 | | 94.6 | 97.7 | | 100 | | 96.8 |  |  |
| Nsp11 | 88.2 | | 88.6 | | 88.3 | 87.9 | | 91.5 | | 89.1 | | 85.4 | | 85.4 | | 85.7 | 91 | | 98.5 | | 92.1 |  |  |
|  | 96 | | 97.3 | | 98.7 | 97.8 | | 97.3 | | 95.5 | | 96 | | 95.5 | | 95.1 | 95.1 | | 98.7 | | 98.7 |  |  |
| Nsp12 | 88.3 | | 87.9 | | 86.8 | 86.8 | | 88.5 | | 88.7 | | 85.1 | | 85.3 | | 90.7 | 89.8 | | 99.1 | | 97.6 |  |  |
|  | 96.1 | | 96.8 | | 95.5 | 95.5 | | 94.8 | | 94.2 | | 92.2 | | 92.2 | | 97.4 | 93.5 | | 99.4 | | 99.4 |  |  |
| ORF2a | 89.8 | | 89.4 | | 89.4 | 89.1 | | 84.4 | | 86.3 | | 85.5 | | 85.1 | | 95.8 | 85.9 | | 98.3 | | 98.4 |  |  |
|  | 89.1 | | 88.7 | | 87.5 | 86.8 | | 85.9 | | 88.3 | | 82.9 | | 81.7 | | 95.3 | 87.2 | | 96.9 | | 96.9 |  |  |
| ORF2b | 93.7 | | 93.2 | | 92.8 | 93.2 | | 89.6 | | 91.4 | | 87.8 | | 86.5 | | 97.7 | 91.4 | | 100 | | 99.1 |  |  |
|  | 94.5 | | 89.2 | | 90.5 | 90.5 | | 91.9 | | 90.5 | | 93.2 | | 98.6 | | 90.5 | 90.5 | | 100 | | 100 |  |  |
| ORF3 | 83 | | 83.8 | | 84.1 | 84.1 | | 78.7 | | 80.3 | | 80.5 | | 79.1 | | 91.1 | 80.5 | | 94.4 | | 95.2 |  |  |
|  | 76.8 | | 77.6 | | 77.6 | 78 | | 74.1 | | 74.9 | | 74.5 | | 72.2 | | 86.3 | 75.3 | | 91 | | 90.2 |  |  |
| ORF4 | 82.5 | | 83.1 | | 81.6 | 82.5 | | 85.1 | | 85.1 | | 86.6 | | 85.3 | | 83.6 | 85.8 | | 92.6 | | 92 |  |  |
|  | 76.4 | | 78.2 | | 77.1 | 78.2 | | 77.7 | | 78.8 | | 79.3 | | 77.7 | | 78.8 | 78.8 | | 84.9 | | 84.4 |  |  |
| ORF5a | 85.1 | | 87.2 | | 84.4 | 81.6 | | 94.9 | | 93.6 | | 91.5 | | 90.1 | | 87.2 | 91.5 | | 99.3 | | 97.9 |  |  |
|  | 83 | | 85.1 | | 80.9 | 80.9 | | 91.3 | | 89.4 | | 93.6 | | 91.5 | | 83 | 87.2 | | 97.9 | | 95.7 |  |  |
| ORF5 | 84.7 | | 86.2 | | 84.2 | 84.1 | | 91.5 | | 91.7 | | 87.6 | | 86.9 | | 83.6 | 90.5 | | 98.8 | | 97.3 |  |  |
|  | 83.6 | | 85.1 | | 84.6 | 83.6 | | 94.5 | | 92 | | 90 | | 88.1 | | 84.6 | 92 | | 97.5 | | 97 |  |  |
| ORF6 | 90.1 | | 87.4 | | 88.8 | 88.4 | | 94.9 | | 93.9 | | 92.8 | | 92.6 | | 88.4 | 95.2 | | 99.8 | | 98.3 |  |  |
|  | 93.1 | | 90.9 | | 92 | 91.4 | | 98.3 | | 96.6 | | 93.1 | | 94.3 | | 92 | 98.3 | | 100 | | 96.6 |  |  |
| ORF7 | 89.8 | | 89.2 | | 88.2 | 88.2 | | 93.5 | | 91.1 | | 91.7 | | 90.6 | | 87.1 | 91 | | 98.1 | | 98.9 |  |  |
|  | 91.1 | | 91.1 | | 89.5 | 89.5 | | 96.8 | | 91.9 | | 92.7 | | 91.9 | | 88.7 | 92.6 | | 97.6 | | 99.2 |  |  |
| 3‘UTR | 90.7 | | 90.1 | | 90.7 | 89.3 | | 97.3 | | 96.7 | | 94.7 | | 94 | | 88.1 | 96 | | 98 | | 97.4 |  |  |
|  | / | | / | | / | / | | / | | / | | / | | / | | / | / | | / | | / |  |  |

**Supplementary Table 4** Nucleotide and amino acid identities of different regions of CHbj2103 genome with other representative PRRSV strains

| **Region** | **Pairwise % Identity (nt/aa)** | | | | | | | | | | | | | | | | | | | | | |
| --- | --- | --- | --- | --- | --- | --- | --- | --- | --- | --- | --- | --- | --- | --- | --- | --- | --- | --- | --- | --- | --- | --- |
|  | **VR2332** | **CH-1a** | | **JXA1** | | **JXA1 P80** | | **NADC30** | | **CHsx1401** | | **NADC34** | | **LNWK130** | | **QYYZ** | | | **15SC3** | **CHbj2101** | **CHbj2102** |  |
| 5‘UTR | 89.8 | | 95.2 | | 96.3 | | 88.7 | | 89.3 | | 88.8 | | 91.4 | | 89.8 | | 94.1 | 96.7 | | 99.5 | 99.5 |  |
|  | / | | / | | / | | / | | / | | / | | / | | / | | / | / | | / |  |  |
| Nsp1α | 89.1 | | 93.1 | | 92.4 | | 93 | | 87.4 | | 86.5 | | 86.9 | | 87.4 | | 92 | 92.4 | | 97 | 96.9 |  |
|  | 95 | | 96.1 | | 96.7 | | 96.7 | | 93.9 | | 93.9 | | 96.1 | | 95.6 | | 96.1 | 96.1 | | 98.3 | 97.8 |  |
| Nsp1β | 84.2 | | 89.2 | | 92.8 | | 92.4 | | 77.7 | | 75.9 | | 79.6 | | 76.7 | | 83.3 | 91.6 | | 88 | 87.8 |  |
|  | 82.3 | | 85.7 | | 92.1 | | 91.6 | | 72.9 | | 70 | | 76.4 | | 72.9 | | 81.8 | 91.6 | | 88.7 | 88.2 |  |
| Nsp2 | 78.6 | | 78.3 | | 79.5 | | 77.6 | | 87.3 | | 84.9 | | 76.9 | | 76.3 | | 75.7 | 89 | | 84.8 | 84.9 |  |
|  | 73.1 | | 72.5 | | 74.1 | | 72.5 | | 82.4 | | 80.8 | | 72.4 | | 71.7 | | 69.2 | 85.4 | | 82.2 | 82.2 |  |
| Nsp3 | 84.5 | | 84.9 | | 85.8 | | 85.7 | | 88.4 | | 89.4 | | 85.5 | | 84.6 | | 79.9 | 89.6 | | 87.2 | 87.1 |  |
|  | 92.2 | | 90.4 | | 90.4 | | 90.4 | | 93 | | 94.3 | | 90.9 | | 90 | | 87 | 94.3 | | 93 | 92.6 |  |
| Nsp4 | 85.3 | | 84.6 | | 84.8 | | 84.5 | | 93.3 | | 99.5 | | 81.5 | | 81.7 | | 82 | 92.8 | | 81.7 | 82 |  |
|  | 93.1 | | 92.2 | | 93.6 | | 93.1 | | 97.1 | | 99.5 | | 92.2 | | 91.7 | | 91.7 | 97.5 | | 91.7 | 92.6 |  |
| Nsp5 | 88.6 | | 90.6 | | 87.3 | | 87.6 | | 93.1 | | 100 | | 83.9 | | 83.7 | | 82.4 | 92.4 | | 81.2 | 82.2 |  |
|  | 90 | | 91.2 | | 89.4 | | 89.4 | | 94.7 | | 100 | | 88.8 | | 87.6 | | 89.4 | 92.4 | | 87.1 | 88.2 |  |
| Nsp6 | 83.3 | | 83.3 | | 83.3 | | 83.3 | | 79.2 | | 79.2 | | 50 | | 100 | | 81.2 | 87.5 | | 81.2 | 83.3 |  |
|  | 93.8 | | 87.5 | | 87.5 | | 87.5 | | 87.5 | | 100 | | 87.5 | | 87.5 | | 87.5 | 93.8 | | 87.5 | 87.5 |  |
| Nsp7 | 85.8 | | 82.9 | | 81.3 | | 81.3 | | 94.2 | | 99.5 | | 83.8 | | 83.7 | | 80.6 | 94.2 | | 80.1 | 79.7 |  |
|  | 88 | | 84.2 | | 83.4 | | 83 | | 94.2 | | 99.6 | | 87.3 | | 87.6 | | 84.2 | 95 | | 83.4 | 83.4 |  |
| Nsp8 | 90.6 | | 89.1 | | 88.4 | | 89.1 | | 93.5 | | 89.9 | | 94.9 | | 99.3 | | 86.2 | 94.2 | | 84.8 | 84.1 |  |
|  | 93.5 | | 93.5 | | 93.5 | | 93.5 | | 95.7 | | 100 | | 97.8 | | 95.7 | | 93.5 | 93.5 | | 89.1 | 89.1 |  |
| Nsp9 | 88.3 | | 88.5 | | 89 | | 88.6 | | 90.9 | | 93.2 | | 87.5 | | 86.7 | | 86.1 | 90.9 | | 94.4 | 94.3 |  |
|  | 96.4 | | 96.4 | | 96.9 | | 97 | | 96.6 | | 98.9 | | 96.1 | | 95.3 | | 95.2 | 96.7 | | 97.2 | 97 |  |
| Nsp10 | 84.2 | | 84.4 | | 84.1 | | 84.2 | | 91.5 | | 92.1 | | 89.3 | | 89.1 | | 84.1 | 92.5 | | 96.4 | 96.4 |  |
|  | 92.3 | | 90.9 | | 91.4 | | 91.8 | | 94.3 | | 95.5 | | 95 | | 95.2 | | 91.4 | 95.5 | | 96.8 | 96.8 |  |
| Nsp11 | 89.2 | | 91.3 | | 89.5 | | 89.7 | | 92.1 | | 89.2 | | 86.5 | | 86.5 | | 86.5 | 93 | | 91.9 | 92.1 |  |
|  | 95.5 | | 96.9 | | 97.3 | | 97.3 | | 96.4 | | 94.6 | | 95.5 | | 95.1 | | 94.6 | 97.8 | | 97.3 | 98.7 |  |
| Nsp12 | 89.2 | | 88.7 | | 87.2 | | 87.2 | | 89 | | 88.7 | | 85.7 | | 86.4 | | 90.9 | 90 | | 97.2 | 97.6 |  |
|  | 96.1 | | 96.8 | | 95.5 | | 95.5 | | 94.8 | | 94.2 | | 92.2 | | 92.2 | | 97.4 | 94.8 | | 98.7 | 99.4 |  |
| ORF2a | 89.9 | | 89.8 | | 89.2 | | 89.2 | | 84.6 | | 86.4 | | 85.7 | | 85.2 | | 96.5 | 86 | | 98.8 | 98.4 |  |
|  | 89.5 | | 89.9 | | 87.9 | | 87.9 | | 86.7 | | 89.5 | | 83.7 | | 82.1 | | 97.3 | 87.9 | | 98.4 | 96.9 |  |
| ORF2b | 92.8 | | 92.3 | | 91.9 | | 92.3 | | 88.7 | | 90.5 | | 87.4 | | 86 | | 96.8 | 90.1 | | 99.1 | 99.1 |  |
|  | 94.5 | | 89.2 | | 90.5 | | 90.5 | | 91.9 | | 90.5 | | 93.2 | | 98.6 | | 90.5 | 90.5 | | 100 | 100 |  |
| ORF3 | 85.8 | | 86.9 | | 87.2 | | 87.5 | | 81.3 | | 82.9 | | 83.1 | | 81.7 | | 94 | 82.4 | | 98.2 | 95.2 |  |
|  | 80.7 | | 83.9 | | 84.3 | | 84.3 | | 80 | | 81.2 | | 79.2 | | 76.9 | | 91.8 | 81.2 | | 97.3 | 90.2 |  |
| ORF4 | 86.4 | | 87.7 | | 86.2 | | 87.2 | | 89.8 | | 89.6 | | 91.1 | | 89.8 | | 88.3 | 90.9 | | 98.7 | 92 |  |
|  | 87.1 | | 89.9 | | 88.8 | | 89.9 | | 87.2 | | 88.8 | | 89.4 | | 87.7 | | 91.6 | 86.6 | | 97.2 | 84.4 |  |
| ORF5a | 85.8 | | 86.5 | | 85.1 | | 82.3 | | 95.7 | | 94.3 | | 92.2 | | 90.8 | | 87.9 | 95 | | 97.2 | 97.9 |  |
|  | 85.1 | | 85.1 | | 80.9 | | 80.9 | | 93.5 | | 91.5 | | 93.6 | | 91.5 | | 87.2 | 93.6 | | 93.6 | 95.7 |  |
| ORF5 | 83.6 | | 85.2 | | 84.1 | | 84.1 | | 91.5 | | 92.2 | | 86.7 | | 86.1 | | 82.9 | 92.2 | | 97.5 | 97.3 |  |
|  | 83.1 | | 85.1 | | 85.1 | | 84.1 | | 95.5 | | 94 | | 90.5 | | 88.1 | | 83.6 | 94.5 | | 99 | 97 |  |
| ORF6 | 89.5 | | 86.9 | | 88.2 | | 87.8 | | 94.3 | | 93.3 | | 92 | | 91.8 | | 88.2 | 94.7 | | 98.1 | 98.3 |  |
|  | 91.4 | | 89.1 | | 90.3 | | 89.7 | | 96 | | 94.3 | | 91.4 | | 92.6 | | 89.7 | 96 | | 96.6 | 96.6 |  |
| ORF7 | 89.8 | | 89.2 | | 88.2 | | 88.2 | | 93.5 | | 91.7 | | 92.2 | | 91.1 | | 86.6 | 92.7 | | 97 | 98.9 |  |
|  | 90.3 | | 90.3 | | 88.7 | | 88.7 | | 96 | | 91.1 | | 91.9 | | 91.1 | | 88.7 | 92.7 | | 96.8 | 99.2 |  |
| 3‘UTR | 91.4 | | 89.4 | | 90 | | 88.7 | | 98 | | 96.7 | | 94.7 | | 94 | | 88.7 | 96.7 | | 98 | 97.4 |  |
|  | / | | / | | / | | / | | / | | / | | / | | / | | / | / | | / | / |  |

**Supplementary Table 5** Information on the whole genomes of all reported QYYZ-like PRRSV strains in China from 1991 to 2021

| **No.** | **Strain** | **Accession no.** | **Year^a^** | **Country** | **Province** |  |
| --- | --- | --- | --- | --- | --- | --- |
| 1 | MD001 | KP998431 | 1991 | China | Taiwan |  |
| 2 | TD1 | KP998425 | 1997 | China | Taiwan |  |
| 3 | TD/TP | KP998422 | 1998 | China | Taiwan |  |
| 4 | Tsai | KP998423 | 1999 | China | Taiwan |  |
| 5 | NT | KP998420 | 2000 | China | Taiwan |  |
| 6 | TY1 | KP998424 | 2000 | China | Taiwan |  |
| 7 | M1 | KP998419 | 2001 | China | Taiwan |  |
| 8 | CH | KP998416 | 2002 | China | Taiwan |  |
| 9 | HK2 | KF287133 | 2003 | China | Hongkong |  |
| 10 | CH8V-J2 | KP998426 | 2003 | China | Taiwan |  |
| 11 | HK6 | KF287135 | 2004 | China | Hongkong |  |
| 12 | HK9 | KF287137 | 2004 | China | Hongkong |  |
| 13 | HK11 | KF287138 | 2004 | China | Hongkong |  |
| 14 | HK15 | KF287142 | 2004 | China | Hongkong |  |
| 15 | HK16 | KF287143 | 2004 | China | Hongkong |  |
| 16 | HL | KP998418 | 2004 | China | Taiwan |  |
| 17 | TD-2 | KP998427 | 2004 | China | Taiwan |  |
| 18 | 17199 | KP998401 | 2005 | China | Taiwan |  |
| 19 | 312 | KP998402 | 2005 | China | Taiwan |  |
| 20 | 310 | KP998413 | 2005 | China | Taiwan |  |
| 21 | Q94-136 | KP998421 | 2005 | China | Taiwan |  |
| 22 | 25934 | KP998412 | 2008 | China | Taiwan |  |
| 23 | 660 | KP998414 | 2009 | China | Taiwan |  |
| 24 | QYYZ | JQ308798 | 2010 | China | Guangdong | |
| 25 | QY2010 | JQ743666 | 2010 | China | Guangdong | |
| 26 | GM2 | JN662424 | 2011 | China | Guangdong | |
| 27 | 338 | KP998404 | 2011 | China | Taiwan | |
| 28 | JM | KP998410 | 2011 | China | Taiwan | |
| 29 | SH1211 | KF678434 | 2012 | China | Shanghai | |
| 30 | 1483 | KP998403 | 2012 | China | Taiwan | |
| 31 | HC120821-SH1 | KP998406 | 2012 | China | Taiwan | |
| 32 | HC120821-SH2 | KP998407 | 2012 | China | Taiwan | |
| 33 | HC120821-LL | KP998408 | 2012 | China | Taiwan | |
| 34 | HC120904-CHYL | KP998409 | 2012 | China | Taiwan | |
| 35 | HC120629 | KP998430 | 2012 | China | Taiwan | |
| 36 | 803 | KP998415 | 2013 | China | Taiwan | |
| 37 | HiNZWQ | KY373215 | 2014 | China | Hainan | |
| 38 | GD1404 | MF124329 | 2014 | China | Guangdong | |
| 39 | FJFS | KP998476 | 2015 | China | Fujian | |
| 40 | HNyc15 | KT945018 | 2015 | China | Henan | |
| 41 | GD-KP | KU978619 | 2015 | China | Guangdong | |
| 42 | GDsg | KX621003 | 2015 | China | Guangdong | |
| 43 | XJzx1-2015 | KX689233 | 2015 | China | Xinjiang | |
| 44 | SDqd1501 | MN642099 | 2015 | China | Shandong | |
| 45 | JX/CH/2016 | KY495780 | 2016 | China | Jiangxi | |
| 46 | SH/CH/2016 | KY495781 | 2016 | China | Shanghai | |
| 47 | GDYDZZZ | KY745901 | 2016 | China | Guangdong | |
| 48 | SCcd16 | MF196905 | 2016 | China | Sichuan | |
| 49 | GDQYQC2 | MF526896 | 2016 | China | Guangdong | |
| 50 | GDZS2016 | MH046843 | 2016 | China | Guangdong | |
| 51 | ZJnb16-2 | MH236426 | 2016 | China | Zhejiang | |
| 52 | SD110-1608 | MK780825 | 2016 | China | Shandong | |
| 53 | HN-YL1711 | MT708500 | 2017 | China | Henan | |
| 54 | FJLIUY-2017 | MG011718 | 2017 | China | Fujian | |
| 55 | FJDJQ-2017 | MG011719 | 2017 | China | Fujian | |
| 56 | FJNP2017 | MH046842 | 2017 | China | Fujian | |
| 57 | SCya17 | MH324400 | 2017 | China | Sichuan | |
| 58 | GZgy17 | MK144542 | 2017 | China | Guizhou | |
| 59 | SDWH27-1710 | MK780824 | 2017 | China | Shandong | |
| 60 | PRRSV2/CN/F1004/2017 | MT416544 | 2017 | China | NA^b^ | |
| 61 | SCya18 | MK144543 | 2018 | China | Sichuan | |
| 62 | LN-DB87 | MN046242 | 2018 | China | Liaoning | |
| 63 | SW2018001-YL | MN401750 | 2018 | China | Taiwan | |
| 64 | PRRSV2/CN/N9185/2018 | MT416542 | 2018 | China | NA^b^ | |
| 65 | GXXNF10-1803 | ON462046 | 2018 | China | Guangxi | |
| 66 | GXXNF53-1805 | ON462047 | 2018 | China | Guangxi | |
| 67 | GDXNF60-1805 | ON462048 | 2018 | China | Guangdong | |
| 68 | GXXNF74-1806 | ON462049 | 2018 | China | Guangxi | |
| 69 | GXXNF78-1806 | ON462050 | 2018 | China | Guangxi | |
| 70 | GDXNF229-1811 | ON462051 | 2018 | China | Guangdong | |
| 71 | HNLCL15-1903 | ON462043 | 2019 | China | Henan | |
| 72 | GXNN202010 | MW561593 | 2020 | China | Guangxi | |
| 73 | PRRSV2/CN/L4/2020 | [OL422822](https://www.ncbi.nlm.nih.gov/nuccore/OL422822) | 2020 | China | Fujian | |
| 74 | GX11045 | OM202902 | 2020 | China | Guangxi | |
| 75 | GX11373 | OM202903 | 2020 | China | Guangxi | |
| 76 | PRRSV/CN/SS0/2020 | ON365556 | 2020 | China | Fujian | |
| 77 | HNTZJ1714-2011 | ON462044 | 2020 | China | Henan | |
| 78 | PRRSV2/CN/L3/2021 | [OL416130](https://www.ncbi.nlm.nih.gov/nuccore/OL416130) | 2021 | China | Fujian | |
| 79 | PRRSV2/CN/SS1/2021 | ON093974 | 2021 | China | Fujian | |
| 80 | GXTZJ2325-2112 | ON462045 | 2021 | China | Guangxi | |
| 81 | CHbj2101^c^ | OP734316 | 2021 | China | Beijing | |
| 82 | CHbj2102^c^ | OP734317 | 2021 | China | Beijing | |
| 83 | CHbj2103^c^ | OP734318 | 2021 | China | Beijing | |

^a^ The year of virus isolation.

^b^ Not available.

^c^ Strains isolated in this study.

**Supplementary Table 6** Recombination information of QYYZ-like PRRSV in 1991-2021

| **No.** | **strain** | **Major parent** | **Minor parent** | **L3 Region1** | | | | **L3 Region2** | | | | **L3 Region3** | | |  |
| --- | --- | --- | --- | --- | --- | --- | --- | --- | --- | --- | --- | --- | --- | --- | --- |
|  |  |  |  | **Left** | **Right** | **Location** | **Left** | | **Right** | **Location** | **Left** | | **Right** | **Location** | |
| 1 | GM2 | L3 (QYYZ) | L5 (VR2332) | 1 | 7462 | 5'UTR-nsp8 | 11093 | | 15558 | nsp11-3'UTR |  | |  |  | |
| 2 | SH1211 | L8 (JXA1) | L3 (QYYZ) | 11697 | 12768 | nsp12-ORF2 | 13819 | | 14472 | ORF5-ORF6 |  | |  |  | |
| 3 | HiNZWQ | L8 (JXA1) | L3 (QYYZ) | 12682 | 12872 | ORF3 |  | |  |  |  | |  |  | |
| 4 | GD1404 | L8 (JXA1 P80) | L3 (QYYZ) | 13511 | 14988 | ORF4-3'UTR |  | |  |  |  | |  |  | |
| 5 | FJFS | L3 (QYYZ) | L8 (JXA1) | 2106 | 15429 | nsp2-3'UTR |  | |  |  |  | |  |  | |
| 6 | GD-KP | L8 (JXA1)+L5 (VR2332) | L3 (QYYZ) | 2201 | 5241 | nsp2-nsp3 | 6341 | | 7441 | nsp5-nsp7 | 11021 | | 15304 | nsp11-3'UTR | |
| 7 | GDsg | L3 (QYYZ) | L8 (JXA1 P80) | 1 | 161 | 5'UTR | 3021 | | 7459 | nsp2-nsp7 | 10601 | | 13101 | nsp10-ORF3 | |
| 8 | XJzx1-2015 | L8 (NB/04) | L3 (QYYZ) | 14014 | 14876 | ORF5-ORF7 |  | |  |  |  | |  |  | |
| 9 | SDqd1501 | L8 (JXA1) | L3 (QYYZ) | 3899 | 6760 | nsp2-nsp8 | 11336 | | 13105 | nsp11-ORF3 | 13827 | | 15172 | ORF5-ORF7 | |
| 10 | JX/CH/2016 | L8 (JXA1) | L3 (QYYZ) | 11861 | 15241 | nsp12-3'UTR |  | |  |  |  | |  |  | |
| 11 | SH/CH/2016 | L8 (JXA1) | L3 (QYYZ) | 11861 | 15089 | nsp12-3'UTR |  | |  |  |  | |  |  | |
| 12 | GDYDZZZ | L8 (JXA1) | L3 (QYYZ) | 11371 | 15077 | nsp12-ORF7 |  | |  |  |  | |  |  | |
| 13 | SCcd16 | L8 (JXA1)+L1 (NADC30) | L3 (FJFS) | 13841 | 15281 | ORF5-3'UTR |  | |  |  |  | |  |  | |
| 14 | GDQYQC2 | L8 (HUN4) | L3 (QYYZ) | 11328 | 13843 | nsp11-ORF5 | 14568 | | 15081 | ORF6-ORF7 |  | |  |  | |
| 15 | GDZS2016 | L8 (JXA1) | L3 (FJFS) | 11983 | 14804 | ORF2-ORF6 |  | |  |  |  | |  |  | |
| 16 | ZJnb16-2 | L8 (JXA1) | L3 (QYYZ) | 11189 | 15509 | ORF2-3'UTR |  | |  |  |  | |  |  | |
| 17 | SD110-1608 | L8 (JXA1) | L3 (QYYZ) | 2889 | 6738 | nsp2-nsp7 | 11329 | | 13067 | nsp11-ORF3 | 13792 | | 15211 | ORF4-ORF7 | |
| 18 | HN-YL1711 | L1 (NADC30)+L8 (JXA1) | L3 (QYYZ) | 12798 | 13058 | ORF2-ORF3 |  | |  |  |  | |  |  | |
| 19 | FJLIUY-2017 | L1 (NADC30)+L5 (BJ-4)+L8 (JXA1) | L3 (QYYZ) | 13857 | 15140 | ORF4-3'UTR |  | |  |  |  | |  |  | |
| 20 | FJDJQ-2017 | L1 (NADC30) | L3 (QYYZ) | 12269 | 14098 | ORF2-ORF5 |  | |  |  |  | |  |  | |
| 21 | FJNP2017 | L8 (JXA1) | L3 (QYYZ) | 11954 | 14931 | ORF2-ORF7 |  | |  |  |  | |  |  | |
| 22 | SCya17 | L8 (JXA1)+L1 (NADC30) | L3 (XJzx1-2015) | 12690 | 14777 | ORF3-ORF5 |  | |  |  |  | |  |  | |
| 23 | GZgy17 | L8 (JXA1) | L3 (QYYZ) | 12054 | 14698 | ORF2-ORF6 |  | |  |  |  | |  |  | |
| 24 | SDWH27-1710 | L3 (HK15) | L8 (JXA1) | 2422 | 15445 | nsp2-3'UTR |  | |  |  |  | |  |  | |
| 25 | PRRSV2/CN/F1004/2017 | L8 (JXA1) | L3 (QYYZ) | 13855 | 15320 | ORF4-3'UTR |  | |  |  |  | |  |  | |
| 26 | SCya18 | L1 (NADC30)+L8 (JXA1) | L3 (QYYZ) | 10928 | 14529 | nsp11-ORF6 |  | |  |  |  | |  |  | |
| 27 | LN-DB87 | L8 (JXA1) | L3 (QYYZ) | 12590 | 12825 | ORF2-ORF3 | 12895 | | 13800 | ORF3-ORF5 | 14231 | | 15295 | ORF5-3'UTR | |
| 28 | PRRSV2/CN/N9185/2018 | L1 (NADC30)+L8 (JXA1) | L3 (QYYZ) | 13382 | 15320 | ORF4-3'UTR |  | |  |  |  | |  |  | |
| 29 | GXXNF10-1803 | L8 (JXA1) | L3 (QYYZ) | 3507 | 7781 | nsp2-nsp8 | 12294 | | 15503 | ORF2-3'UTR |  | |  |  | |
| 30 | GXXNF53-1805 | L8 (JXA1) | L3 (QYYZ) | 12685 | 15291 | ORF3-3'UTR |  | |  |  |  | |  |  | |
| 31 | GDXNF60-1805 | L8 (JXA1) | L3 (QYYZ) | 12541 | 15316 | ORF3-3'UTR |  | |  |  |  | |  |  | |
| 32 | GXXNF74-1806 | L3 (QYYZ) | L8 (JXA1) | 2339 | 6924 | nsp2-nsp7 | 8969 | | 15232 | nsp9-3'UTR |  | |  |  | |
| 33 | GXXNF78-1806 | L8 (JXA1) | L3 (QYYZ) | 11987 | 12851 | ORF2-ORF3 | 13673 | | 15266 | ORF5-3'UTR |  | |  |  | |
| 34 | GDXNF229-1811 | L3 (QYYZ) | L8 (JXA1) | 2285 | 6317 | nsp2-nsp5 | 8812 | | 15230 | nsp9-3'UTR |  | |  |  | |
| 35 | HNLCL15-1903 | L1 (NADC30) | L3 (SH/CH/2016) | 1 | 1659 | 5'UTR-nsp2 | 10531 | | 14132 | nsp10-ORF6 |  | |  |  | |
| 36 | GXNN202010 | L8 (JXA1) | L3 (QYYZ) | 12221 | 15318 | ORF2-3'UTR |  | |  |  |  | |  |  | |
| 37 | PRRSV2/CN/L4/2020 | L8 (JXA1)+L1 (NADC30) | L3 (QYYZ) | 11401 | 15411 | nsp11-3'UTR |  | |  |  |  | |  |  | |
| 38 | GX11045 | L8 (TJ ) | L3 (QYYZ) | 11337 | 15582 | nsp11-3'UTR |  | |  |  |  | |  |  | |
| 39 | GX11373 | L8 (TJ+TJbd14-1) | L3 (QYYZ) | 13779 | 14414 | ORF5 |  | |  |  |  | |  |  | |
| 40 | PRRSV/CN/SS0/2020 | L8 (HUN4)+L1 (NADC30) | L3 (QYYZ) | 11788 | 15411 | nsp12-3'UTR |  | |  |  |  | |  |  | |
| 41 | HNTZJ1714-2011 | L8 (JXA1)+L1 (NADC30) | L3 (QYYZ) | 11668 | 15011 | ORF2-3'UTR |  | |  |  |  | |  |  | |
| 42 | PRRSV2/CN/L3/2021 | L8 (JXA1)+L1 (NADC30) | L3 (FJFS) | 1845 | 5390 | nsp2-nsp3 |  | |  |  |  | |  |  | |
| 43 | PRRSV2/CN/SS1/2021 | L8 (HUN4)+L1 (NADC30) | L3 (FJFS) | 13227 | 15411 | ORF3-3'UTR |  | |  |  |  | |  |  | |
| 44 | GXTZJ2325-2112 | L3 (QYYZ) | L8 (JXA1)+L1 (NADC30) | 5268 | 15017 | nsp4-3'UTR |  | |  |  |  | |  |  | |
| 45 | CHbj2101^a^ | L1 (15LN3) | L3 (QYYZ) | 12001 | 13521 | ORF2-ORF4 |  | |  |  |  | |  |  | |
| 46 | CHbj2102^a^ | L1 (15LN3) | L3 (QYYZ) | 12001 | 13521 | ORF2-ORF4 |  | |  |  |  | |  |  | |
| 47 | CHbj2103^a^ | L1 (15SC3) | L3 (QYYZ) | 11921 | 13541 | ORF2-ORF4 |  | |  |  |  | |  |  | |

^a^ Strains isolated in this study.
